# Kohdista: an efficient method to index and query possible Rmap alignments

**DOI:** 10.1186/s13015-019-0160-9

**Published:** 2019-12-12

**Authors:** Martin D. Muggli, Simon J. Puglisi, Christina Boucher

**Affiliations:** 1 Department of Computer Science, Colorado State University, Fort Collins, CO USA; 2 Department of Computer Science, University of Helsinki, Helsinki, Finland; 3 Computer & Information Science & Engineering, University of Florida, Gainesville, FL USA

**Keywords:** Optical mapping, Index based data structures, FM-index, Graph algorithms

## Abstract

**Background:**

Genome-wide optical maps are ordered high-resolution restriction maps that give the position of occurrence of restriction cut sites corresponding to one or more restriction enzymes. These genome-wide optical maps are assembled using an overlap-layout-consensus approach using raw optical map data, which are referred to as Rmaps. Due to the high error-rate of Rmap data, finding the overlap between Rmaps remains challenging.

**Results:**

We present Kohdista, which is an index-based algorithm for finding pairwise alignments between single molecule maps (*Rmaps*). The novelty of our approach is the formulation of the alignment problem as automaton path matching, and the application of modern index-based data structures. In particular, we combine the use of the Generalized Compressed Suffix Array (GCSA) index with the wavelet tree in order to build Kohdista. We validate Kohdista on simulated *E. coli* data, showing the approach successfully finds alignments between Rmaps simulated from overlapping genomic regions.

**Conclusion:**

we demonstrate Kohdista is the only method that is capable of finding a significant number of high quality pairwise Rmap alignments for large eukaryote organisms in reasonable time.

## Background

There is a current resurgence in generating diverse types of data, to be used alone or in concert with short read data, in order to overcome the limitations of short read data. Data from an optical mapping system [1] is one such example and has itself become more practical with falling costs of high-throughput methods. For example, the current BioNano Genomics Irys System requires one week and $1000 USD to produce the Rmap data for an average size eukaryote genome, whereas, it required $100,000 and 6 months in 2009 [2]. These technological advances and the demonstrated utility of optical mapping in genome assembly [3–7] have driven several recent tool development efforts [8–10].

Genome-wide optical maps are ordered high-resolution restriction maps that give the position of occurrence of restriction cut sites corresponding to one or more restriction enzymes. These genome-wide optical maps are assembled using an overlap-layout-consensus approach using raw optical map data, which are referred to as *Rmaps*. Hence, Rmaps are akin to reads in genome sequencing. In addition, to the the inaccuracies in the fragment sizes, there is the possibility of cut sites being spuriously added or deleted; which makes the problem of finding pairwise alignments between Rmaps challenging. To date, however, there is no efficient, non-proprietary method for finding pairwise alignments between Rmaps, which is the first step in assembling genome-wide maps.

Several existing methods are superficially applicable to Rmap pairwise alignments but all programs either struggle to scale to even moderate size genomes or require significant further adaptation to the problem. Several methods exhaustively evaluate all pairs of Rmaps using dynamic programming. One of these is the method of Valouev et al. [11], which is capable of solving the problem exactly but requires over 100,000 CPU hours to compute the alignments for rice [12]. The others are SOMA [13] and MalignerDP [10] which are designed only for semi-global alignments instead of overlap alignments, which are required for assembly.

Other methods reduce the number of map pairs to be individually considered by initially finding seed matches and then extending them through more intensive work. These include OMBlast [9], OPTIMA [8], and MalignerIX [10]. These, along with MalignerDP, were designed for a related alignment problem of aligning consensus data but cannot consistently find high quality Rmap pairwise alignments in reasonable time as we show later. This is unsurprising since these methods were designed for either already assembled optical maps or in silico digested sequence data for one of their inputs, both having a lower error rate than Rmap data. In addition, Muggli et al. [14] presented a method called Twin, which aligns assembled contigs to a genome-wide optimal map. Twin varies from these previous methods in that it is unable to robustly find alignments between pairs of Rmaps due to the presence of added or missing cut-sites.

In this paper, we present a fast, error-tolerant method for performing pairwise Rmap alignment that makes use of a novel FM-index based data structure. Although the FM-index can naturally be applied to short read alignment [15, 16], it is nontrivial to apply it to Rmap alignment. The difficulty arises from: (1) the abundance of missing or false cut sites, (2) the fragment sizes require inexact fragment-fragment matches (e.g. 1547 bp and 1503 bp represent the same fragment), (3) the Rmap sequence alphabet consists of all unique fragment sizes and is so extremely large (e.g., over 16,000 symbols for the goat genome). The second two challenges render inefficient the standard FM-index backward search algorithm, which excels at exact matching over small alphabets since each step of the algorithm extends the search for a query string by a single character c. If the alphabet is small (say DNA alphabet) then a search for other symbols of the alphabet other than c can be incorporated without much cost to the algorithm’s efficiency. Yet, if the alphabet is large enough this exhaustive search becomes impractical. The wavelet tree helps to remedy this problem. It allows efficiently answering queries of the form: find all symbols that allow extension of the backward search by a single character, where the symbol is within the range $[\alpha _1 \ldots \alpha _k]$ and where $\alpha _1$ and $\alpha _k$ are symbols in the alphabet such that $\alpha _1\le \alpha _k$ [17]. In the case of optical mapping data, the alphabet is all fragment sizes. Thus, Muggli et al. [14] showed that constructing the FM-index and wavelet tree from this input can allow for sizing error to be account for by replacing each query in the FM index backward search algorithm with a range query supported by the wavelet tree, i.e., if the fragment size in the query string is *x* then the wavelet tree can support queries of the form: find all fragment sizes that allow extension of the backward search by a single fragment, where the fragment size in the range $[x - y, x + y]$ occur, where *y* is a threshold on the error tolerance.

Muggli et al. [14] demonstrated that the addition of the wavelet tree can remedy the first two problems, i.e., sizing error and alphabet size, but the first and most-notable challenge requires a more complex index-based data structure. The addition of the wavelet tree to the FM-index is not enough to allow for searches that are robust to inserted and deleted cut sites. To overcome the challenge of having added or deleted cut sites while still accommodating the other two challenges, we develop Kohdista, an index-based Rmap alignment program that is capable of finding all pairwise alignments in large eukaryote organisms.

We first abstract the problem to that of approximate-path matching in a directed acyclic graph (DAG). The Kohdista method then indexes a set of Rmaps represented as a DAG, using a modified form of the *generalized compressed suffix array (GCSA)*, which is a variant of the FM-index developed by Sirén et al. [18]. Hence, the constructed DAG, which is stored using the GCSA, stores all Rmaps, along with all variations obtained by considering all speculative added and deleted cut sites. The GCSA stores the DAG in a manner such that paths in DAG may be queried efficiently. If we contrast this to naïve automaton implementations, the GCSA has two advantages: it is space efficient, and it allows for efficient queries. Lastly, we demonstrate that challenges posed by the inexact fragment sizes and alphabet size can be overcome, specifically in the context of the GCSA, via careful use of a wavelet tree [17], and via using statistical criteria to control the quality of the discovered alignments.

Next, we point out some practical considerations concerning Kohdista. First, we note that Kohdista can be easily parallelized since once the GCSA is constructed from the Rmap data, it can be queried in parallel on as many threads as there are Rmaps to be queried. Next, in this paper, we focus on finding all pairwise alignments that satisfy some statistical constraints—whether they be global or local alignments. Partial alignments can be easily obtained by considering the prefix or suffix of the query Rmap and relaxing the statistical constraint.

We verify our approach on simulated *E. coli* Rmap data by showing that Kohdista achieves similar sensitivity and specificity to the method of Valouev et al. [12], and with more permissive alignment acceptance criteria 90% of Rmap pairs simulated from overlapping genomic regions. We also show the utility of our approach on larger eukaryote genomes by demonstrating that existing published methods require more than 151 h of CPU time to find all pairwise alignments in the plum Rmap data; whereas, Kohdista requires 31 h. Thus, we present the first fully-indexed method capable of finding all match patterns in the pairwise Rmap alignment problem.

### Preliminaries and definitions

Throughout we consider a string (or sequence) $S = S[1 \ldots n] = S[1]S[2] \ldots S[n]$ of $|S| = n$ symbols drawn from the alphabet $[1 \ldots \sigma ]$. For $i=1, \ldots ,n$ we write *S*[*i*…*n*] to denote the *suffix* of *S* of length $n-i+1$, that is $S[i \ldots n] = S[i]S[i+1] \ldots S[n]$, and *S*[1…*i*] to denote the *prefix* of *S* of length *i*. *S*[*i*…*j*] is the *substring* $S[i]S[i+1] \ldots S[j]$ of *S* that starts at position *i* and ends at *j*. Given a sequence *S*[1, *n*] over an alphabet $\Sigma = \{1, \ldots ,\sigma \}$, a character $c \in \Sigma$, and integers *i*,*j*, ${\textsf {rank}}_c(S,i)$ is the number of times that *c* appears in *S*[1, *i*], and ${\textsf {select}}_c(S,j)$ is the position of the *j*-th occurrence of *c* in *S*. We remove *S* from the functions when it is implicit from the context.

#### Overview of optical mapping

From a computer science viewpoint, restriction mapping (by optical or other means) can be seen as a process that takes in two sequences: a genome ${\mathsf {A}}[1,n]$ and a restriction enzyme’s restriction sequence ${\mathsf {B}}[1,b]$, and produces an array (sequence) of integers ${\textsf {C}}$, the *genome restriction map*, which we define as follows. First define the array of integers ${\textsf {C}}[1,m]$ where ${\textsf {C}}[i] = j$ if and only if ${\mathsf {A}}[j \ldots j+b] = {\mathsf {B}}$ is the *i*th occurrence of ${\mathsf {B}}$ in ${\mathsf {A}}$. Then ${\textsf {R}}[i] = ({\textsf {C}}[i]-{\textsf {C}}[i-1])$, with ${\textsf {R}}[1] = {\textsf {C}}[1]-1$. In words, ${\textsf {R}}$ contains the distance between occurrences of ${\mathsf {B}}$ in ${\mathsf {A}}$. For example, if we let ${\mathsf {B}}$ be act and ${\mathsf {A}}= {\texttt {atacttactggactactaaact}}$ then we would have ${\textsf {C}}= 3,7,12,15,20$ and ${\textsf {R}}= 2,4,5,3,5$. In reality, ${\textsf {R}}$ is a consensus sequence formed from millions of erroneous Rmap sequences. The optical mapping system produces millions of Rmaps for a single genome. It is performed on many cells of an organism and for each cell there are thousands of Rmaps (each at least 250 Kbp in length in publicly available data). The Rmaps are then assembled to produce a genome-wide optical map. Like the final ${\textsf {R}}$ sequence, each Rmap is an array of lengths—or fragment sizes—between occurrences of ${\mathsf {B}}$ in ${\mathsf {A}}$.

There are three types of errors that an Rmap (and hence with lower magnitude and frequency, also the consensus map) can contain: (1) missing and false cuts, which are caused by an enzyme not cleaving at a specific site, or by random breaks in the DNA molecule, respectively; (2) missing fragments that are caused by *desorption*, where small ($< 1$ Kbp ) fragments are lost and so not detected by the imaging system; and (3) inaccuracy in the fragment size due to varying fluorescent dye adhesion to the DNA and other limitations of the imaging process. Continuing again with the example above where ${\textsf {R}}= 2,4,5,3,5$ is the error-free Rmap: an example of an Rmap with the first type of error could be ${\textsf {R}}' = 6,5,3,5$ (the first cut site is missing so the fragment sizes 2, and 4 are summed to become 6 in ${\textsf {R}}'$); an example of an Rmap with the second type of error would be ${\textsf {R}}'' = 2,4,3,5$ (the third fragment is missing); and lastly, the third type of error could be illustrated by ${\textsf {R}}''' = 2,4,7,3,5$ (the size of the third fragment is inaccurately given).

#### Frequency of errors

In the optical mapping system, there is a 20% probability that a cut site is missed and a 0.15% probability of a false break per Kbp, i.e., error type (1) occurs in a fragment. Popular restriction enzymes in optical mapping experiments recognize a 6 bp sequence giving an expected cutting density of 1 per 4096 bp. At this cutting density, false breaks are less common than missing restriction sites (approx. $0.25 * .2 = .05$ for missing sites vs. 0.0015 for false sites per bp). The error in the fragment size is normally distributed with a mean of 0 bp, and a variance of $\ell \sigma ^2$, where $\ell$ is equal to the fragment length and $\sigma = .58$ kbp [11].

#### Suffix arrays, BWT and backward search

The suffix array [19] ${\textsf {SA}}_{{\mathsf {X}}}$ (we drop subscripts when they are clear from the context) of a sequence ${\mathsf {X}}$ is an array ${\textsf {SA}}[1 \ldots n]$ which contains a permutation of the integers [1...*n*] such that ${\mathsf {X}}[{\textsf {SA}}[1] \ldots n]< {\mathsf {X}}[{\textsf {SA}}[2] \ldots n]< \cdots < {\mathsf {X}}[{\textsf {SA}}[n] \ldots n].$ In other words, ${\textsf {SA}}[j] = i$ iff ${\mathsf {X}}[i \ldots n]$ is the $j{\text{ th }}$ suffix of ${\mathsf {X}}$ in lexicographic order. For a sequence ${\mathsf {Y}}$, the ${\mathsf {Y}}$-interval in the suffix array ${\textsf {SA}}_{{\mathsf {X}}}$ is the interval ${\textsf {SA}}[s \ldots e]$ that contains all suffixes having ${\mathsf {Y}}$ as a prefix. The ${\mathsf {Y}}$-interval is a representation of the occurrences of ${\mathsf {Y}}$ in ${\mathsf {X}}$. For a character *c* and a sequence ${\mathsf {Y}}$, the computation of $c{\mathsf {Y}}$-interval from ${\mathsf {Y}}$-interval is called a *left extension*.

The Burrows–Wheeler Transform $${\textsf {BWT}}[1 \ldots n]$$ is a permutation of $${\mathsf {X}}$$ such that $${\textsf {BWT}}[i] = {\mathsf {X}}[{\textsf {SA}}[i]-1]$$ if $${\textsf {SA}}[i]>1$$ and $ otherwise [20]. We also define $${\textsf {LF}}[i] = j$$ iff $${\textsf {SA}}[j] = {\textsf {SA}}[i]-1$$, except when $${\textsf {SA}}[i] = 1$$, in which case $${\textsf {LF}}[i] = I$$, where $${\textsf {SA}}[I] = n$$. Ferragina and Manzini [21] linked $${\textsf {BWT}}$$ and $${\textsf {SA}}$$ in the following way. Let $${\textsf {C}}[c]$$, for symbol *c*, be the number of symbols in $${\mathsf {X}}$$ lexicographically smaller than *c*. The function $${\textsf {rank}}({\mathsf {X}},c,i)$$, for sequence $${\mathsf {X}}$$, symbol *c*, and integer *i*, returns the number of occurrences of *c* in $${\mathsf {X}}[1 \ldots i]$$. It is well known that $${\textsf {LF}}[i] = {\textsf {C}}[{\textsf {BWT}}[i]] + {\textsf {rank}}({\textsf {BWT}},{\textsf {BWT}}[i],i)$$. Furthermore, we can compute the left extension using $${\textsf {C}}$$ and $${\textsf {rank}}$$. If $${\textsf {SA}}[s \ldots e]$$ is the $${\mathsf {Y}}$$-interval, then $${\textsf {SA}}[{\textsf {C}}[c]+{\textsf {rank}}({\textsf {BWT}},c,s),{\textsf {C}}[c]+{\textsf {rank}}({\textsf {BWT}},c,e)]$$ is the $$c{\mathsf {Y}}$$-interval. This is called *backward search*, and a data structure supporting it is called an *FM-index* [21].

To support rank queries in backward search, a data structure called a *wavelet tree* can be used [17]. It occupies $n\log \sigma + o(n\log \sigma )$ bits of space and supports ${\textsf {rank}}$ queries in $O(\log \sigma )$ time. Wavelet trees also support a variety of more complex queries on the underlying string efficiently. We refer the reader to Gagie et al. [17] for a more thorough discussion of wavelet trees. One such query we will use in this paper is to return the set *X* of distinct symbols occurring in *S*[*i*, *j*], which takes $O(|X|\log \sigma )$ time.

### The pairwise Rmap alignment problem

The pairwise Rmap alignment problem aims to align one Rmap (the *query*) ${\textsf {R}}_q$ against the set of all other Rmaps in the dataset (the *target*). We denote the target database as ${\textsf {R}}_1 \ldots {\textsf {R}}_n$, where each ${\textsf {R}}_i$ is a sequence of $m_i$ fragment sizes, i.e, ${\textsf {R}}_i = [f_{i1}, \ldots , f_{im_i}]$. An alignment between two Rmaps is a relation between them comprising groups of zero or more consecutive fragment sizes in one Rmap associated with groups of zero or more consecutive fragments in the other. For example, given ${\textsf {R}}_i = [4, 5, 10, 9, 3]$ and ${\textsf {R}}_j = [10, 9, 11]$ one possible alignment is $\{[4,5], [10]\}, \{ [10], [9]\}, \{[9], [11]\}, \{[3], []\}$. A group may contain more than one fragment (e.g. [4, 5]) when the restriction site delimiting the fragments is absent in the corresponding group of the other Rmap (e.g [10]). This can occur if there is a false restriction site in one Rmap, or there is a missing restriction site in the other. Since we cannot tell from only two Rmaps which of these scenarios occurred, for the purpose of our remaining discussion it will be sufficient to consider only the scenario of missed (undigested) restriction sites.

## Implementation

We now describe the algorithm behind Kohdista. Three main insights enable our index-based aligner for Rmap data: (1) abstraction of the alignment problem to a finite automaton; (2) use of the GCSA for storing and querying the automaton; and (3) modification of backward search to use a wavelet tree in specific ways to account for the Rmap error profile.

### Finite automaton

Continuing with the example in the background section, we want to align ${\textsf {R}}' = 6,5,3,5$ to ${\textsf {R}}''' = 2,4,7,3,5$ and vice versa. To accomplish this we cast the Rmap alignment problem to that of matching paths in a finite automaton. A finite automaton is a directed, labeled graph that defines a *language*, or a specific set of sequences composed of vertex labels. A sequence is recognized by an automaton if it contains a matching path: a consecutive sequence of vertex labels equal to the sequence. We represent the target Rmaps as an automaton and the query as a path in this context.

#### Backbone

**Fig. 1:**
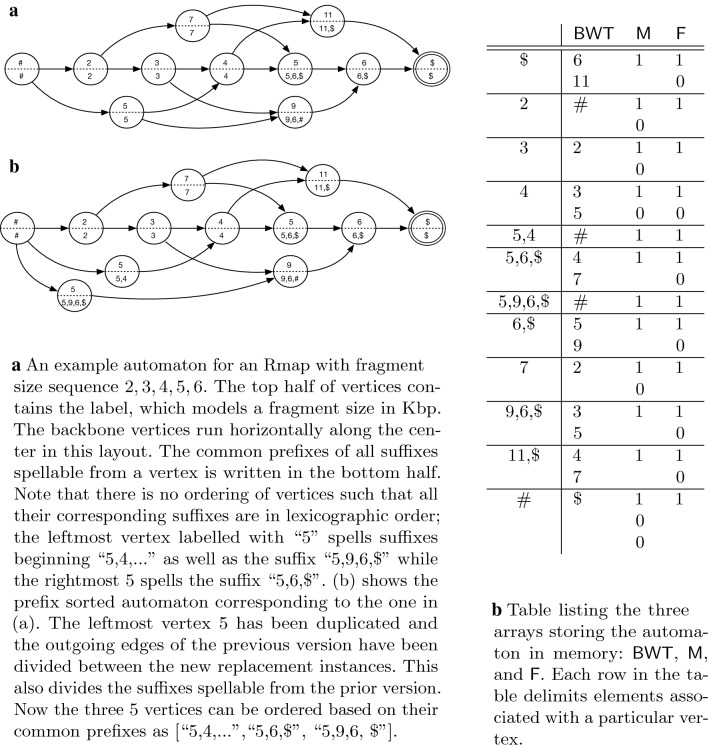
Example automata and corresponding memory representation

The automaton for our target Rmaps can be constructed as follows. First, we concatenate the $${\textsf {R}}_1 \ldots {\textsf {R}}_n$$ together into a single sequence with each Rmap separated by a special symbol which will not match any query symbol. Let $${\textsf {R}}^*$$ denote this concatenated sequence. Hence, $${\textsf {R}}^* = [f_{11}, \ldots ,f_{1m_1}, \ldots , f_{n1}, \ldots ,f_{nm_n}]$$. Then, construct an initial finite automaton $${\mathsf {A}}= (V, E)$$ for $${\textsf {R}}^*$$ by creating a set of vertices $$v^i_1 \ldots v^i_m$$, one vertex per fragment for a total of $$|{\textsf {R}}^*|$$ vertices and each vertex is labeled with the length its corresponding fragment. Edges are then added connecting vertices representing consecutive pairs of elements in $${\textsf {R}}^*$$. Also, introduce to $${\mathsf {A}}$$ a *starting vertex* $$v_1$$ labeled with # and a *final vertex* $$v_f$$ labeled with the character $. All other vertices in $${\mathsf {A}}$$ are labeled with integral values. This initial set of vertices and edges is called the *backbone*. The backbone by itself is only sufficient for finding alignments with no missing cut sites in the query. The backbone of an automaton constructed for a set containing $${\textsf {R}}'$$ and $${\textsf {R}}''$$ would be #, 6, 5, 3, 5, 999, 2, 4, 3, 5$, using 999 as an unmatchable value. Next, extra vertices (“skip vertices”) and extra edges are added to $${\mathsf {A}}$$ to allow for the automaton to accept all valid queries. Figure 1a illustrates the construction of $${\mathsf {A}}$$ for a single Rmap with fragment sizes 2, 3, 4, 5, 6.

#### Skip vertices and skip edges

We introduce extra vertices labeled with *compound fragments* to allow missing cut sites (first type of error) to be taken into account in querying the target Rmaps. We refer to these as *skip vertices* as they provide alternative path segments which skip past two or more backbone vertices. Thus, we add a *skip vertex* to ${\mathsf {A}}$ for every pair of consecutive vertices in the backbone, as well as every triple of consecutive vertices in the backbone, and label these vertices as the sum of the corresponding vertices. For example, vertex labeled with 7 connecting 2 and 5 in 1a is an example of a skip vertex. Likewise, 5, 9, 11 are other skip vertices. Skip vertices corresponding to a pair of vertices in the backbone would correspond to a single missing cut-site and similarly, skip vertices corresponding to a trip of vertices in the backbone correspond to two consecutive missing cut-sites. The probability of more than two consecutive missing cut-sites is negligible [11], and thus, we do not consider more than pairs or triples of vertices. Finally, we add *skip edges* which provide paths around vertices with small labels in the backbone. These allow allow for desorption (the second type of error) to be taken into account in querying ${\textsf {R}}^*$.

### Generalized compressed suffix array

We index the automaton with the GCSA for efficient storage and path querying. The GCSA is a generalization of the FM-index for automata. We will explain the GCSA by drawing on the definition of the more widely-known FM-index. As stated in the background section, the FM-index is based on the deep relationship between the ${\textsf {SA}}$ and the ${\textsf {BWT}}$ data structures of the input string ${\mathsf {X}}$. The ${\textsf {BWT}}$ of an input string is formed by sorting all characters of the string by the lexicographic order of the suffix immediately following each character. The main properties the FM-index exploits in order to perform queries efficiently are (a) ${\textsf {BWT}}[i] = {\mathsf {X}}[{\textsf {SA}}[i]-1]$; and (b) given that ${\textsf {SA}}[i] = j$, and ${\textsf {C}}[c]$ gives the position of the first suffix in ${\textsf {SA}}$ prefixed with character *c*, then using small auxiliary data structures we can quickly determine $k = {\textsf {C}}[{\textsf {BWT}}[i]] + {\textsf {rank}}({\textsf {BWT}},{\textsf {BWT}}[i],i)$, such that ${\textsf {SA}}[k] = j-1$. The first of these properties is simply the definition of the ${\textsf {BWT}}$. The second is, because the symbols of ${\mathsf {X}}$ occur in the same order in both the single character prefixes in the suffix array and in the ${\textsf {BWT}}$, given a set of sorted suffixes, prepending the same character onto each suffix does not change their order. Thus, if we consider all the suffixes in a range of ${\textsf {SA}}$ which are preceded by the same symbol *c*, that subset will appear in the same relative order in ${\textsf {SA}}$: as a contiguous subinterval of the interval that contains all the suffixes beginning with *c*. Hence, by knowing where the position of the internal in ${\textsf {SA}}$ corresponding to a symbol, and the ${\textsf {rank}}$ of an instance of that symbol, we can identify the ${\textsf {SA}}$ position beginning with that instance from its position in ${\textsf {BWT}}$. A rank data structure over the ${\textsf {BWT}}$ constitutes a sufficient compressed index of the suffix array needed for traversal.

To generalize the FM-index to automata from strings, we need to efficiently store the vertices and edges in a manner such that the FM-index properties still hold, allowing the GCSA to support queries efficiently. An FM-index’s compressed suffix array for a string *S* encodes a relationship between each suffix *S* and its left extension. Hence, this suffix array can be generalized to edges in a graph that represent a relationship between vertices. The compressed suffix array for a string is a special case where the vertices are labeled with the string’s symbols in a non-branching path.

#### Prefix-sorted automata

Just as backward search for strings is linked to suffix sorting, backward searching in the BWT of the automaton requires us to be able to sort the vertices (and a set of the paths) of the automaton in a particular way. This property is called *prefix-sorted* by Sirén et al. [18]. Let $A = (V,E)$ be a finite automaton, let $v_{|V|}$ denote its terminal vertex, and let $v \in V$ be a vertex. We say *v* is *prefix-sorted* by prefix *p*(*v*) if the labels of all paths from *v* to $v_{|V|}$ share a *common prefix* *p*(*v*), and no path from any other vertex $u \ne v$ to $v_{|V|}$ has *p*(*v*) as a prefix of its label. Automaton *A* is prefix-sorted if all vertices are prefix-sorted. See Fig. 1a for an example of a non-prefix sorted automaton and a prefix sorted automaton. A non-prefix sorted automaton can be made prefix sorted through a process of duplicating vertices and their incoming edges but dividing their outgoing edges between the new instances. We refer the reader to Sirén et al. [18]) for a more thorough explanation of how to transform a non-prefix sorted automaton to a prefix-sorted one.

Clearly, the prefixes *p*(*v*) allow us to sort the vertices of a prefix-sorted automaton into lexicographical order. Moreover, if we consider the list of outgoing edges (*u*, *v*), sorted by pairs (*p*(*u*), *p*(*v*)), they are also sorted by the sequences $\ell (u)p(v)$, where $\ell (u)$ denotes the label of vertex *u*. This dual sortedness property allows backward searching to work over the list of vertex labels (sorted by *p*(*v*)) in the same way that is does for the symbols of a string ordered by their following suffixes in normal backward search for strings.

Each vertex has a set of one or more preceding vertices and therefore, a set of predecessor labels in the automaton. These predecessor label sets are concatenated to form the ${\textsf {BWT}}$. The sets are concatenated in the order defined by the above mentioned lexicographic ordering of the vertices. Each element in ${\textsf {BWT}}$ then denotes an edge in the automaton. Another bit vector, ${\textsf {F}}$, marks a ‘1’ for the first element of ${\textsf {BWT}}$ corresponding to a vertex and a ‘0’ for all subsequent elements in that set. Thus, the predecessor labels, and hence the associated edges, for a vertex with rank *r* are ${\textsf {BWT}}[{\textsf {select}}_1({\textsf {F}},r) \ldots {\textsf {select}}_1({\textsf {F}},r+1)]$. Another array, ${\textsf {M}}$, stores the outdegree of each vertex and allows the set of vertex ranks associated with a ${\textsf {BWT}}$ interval to be found using ${\textsf {rank}}()$ queries.

### Exact matching: GCSA backward search

Exact matching with the GCSA is similar to the standard FM-index backward search algorithm. As outlined in the background section, FM-index backward search proceeds by finding a succession of lexicographic ranges that match progressively longer suffixes of the query string, starting from the rightmost symbol of the query. The search maintains two items—a lexicographic range and an index into the query string—and the property that the path prefix associated with the lexicographic range is equal to the suffix of the query marked by the query index. Initially, the query index is at the rightmost symbol and the range is [1…*n*] since every path prefix matches the empty suffix. The search continues using GCSA’s backward search step function, which takes as parameters the next symbol (to the left) in the query (i.e. fragment size in ${\textsf {R}}_q$) and the current range, and returns a new range. The query index is advanced leftward after each backward search step. In theory, since the current range corresponds to a consecutive range in the ${\textsf {BWT}}$, the backward search could use ${\textsf {select}}()$ queries on the bit vector ${\textsf {F}}$ (see above) to determine all the edges adjacent to a given vertex and then two FM-index ${\textsf {LF}}()$ queries are applied to the limits of the current range to obtain the new one. GCSA’s implementation uses one succinct bit vector per alphabet symbol to encode which symbols precede a given vertex instead of ${\textsf {F}}$. Finally, this new range, which corresponds to a set of edges, is mapped back to a set of vertices using ${\textsf {rank}}()$ on the ${\textsf {M}}$ bit vector.

### Inexact matching: modified GCSA backward search

We modified GCSA backward search in the following ways. First, we modified the search process to combine consecutive fragments into compound fragments in the query Rmap in order to account for erroneous cut-sites. Secondly, we added and used a wavelet tree in order to allow efficient retrieval of substitution candidates to account for sizing error. Lastly, we introduced backtracking to allow aligning Rmaps in the presence of multiple alternative size substitutions candidates as well as alternative compound fragments for each point in the query. We now discuss these modifications in further detail below.

To accommodate possible false restriction sites that are present in the query Rmap, we generate compound fragments by summing pairs and triples of consecutive query fragment sizes. This summing of multiple consecutive query fragments is complementary to the skip vertices in the target automaton which accommodate false restriction sites in the target. We note for each query Rmap there will be multiple combinations of compound fragments generated.

Next, in order to accommodate possible sizing error in the Rmap data, we modified the backward search by adding and using a wavelet tree in our query of the GCSA. The original implementation of the GCSA does not construct or use the wavelet tree. Although it does consider alignments containing mismatches, it is limited to small alphabets (e.g., DNA alphabet), which do not necessitate the use of the wavelet tree. Here, the alphabet size is all possible fragment sizes. Thus, we construct the wavelet tree in addition to the GCSA. Then when aligning fragment *f* in the query Rmap, we determine the set of candidate fragment sizes that are within some error tolerance of *f* by enumerating the distinct symbols in the currently active backward search range of the ${\textsf {BWT}}$ using the wavelet tree algorithm of Gagie et al. [17]. As previously mentioned, this use of the wavelet tree also exists in the Twin [14] but is constructed and used in conjunction with an FM-index. We used the SDSL-Lite library by Gog et al. [22] to construct and store the GCSA.

Finally, since there may be multiple alternative size compatible candidates in the ${\textsf {BWT}}$ interval of ${\textsf {R}}^*$ for a compound fragment and multiple alternative compound fragments generated at a given position in query Rmap, we add backtracking to backward search so each candidate alignment is evaluated. We note that this is akin to the use of backtracking algorithms in short read alignment [15, 16]. Thus, for a given compound fragment size *f* generated from ${\textsf {R}}_q$, every possible candidate fragment size, $f'$, that can be found in ${\textsf {R}}^*$ in the range $f - t \ldots f + t$ and in the interval $s \ldots e$ (of the ${\textsf {BWT}}$ of ${\textsf {R}}^*$) for some tolerance *t* is considered as a possible substitute in the backward search.

Thus, to recap, when attempting to align each query Rmap, we consider every possible combination of compound fragments and use the wavelet tree to determine possible candidate matches during the backward search. There are potentially a large number of possible candidate alignments—for efficiency, these candidates are pruned by evaluating the alignment during each step of the search relative to statistical models of the expected error in the data. We discuss this pruning in the next subsection.

### Pruning the search

Alignments are found by incrementally extending candidate partial alignments (paths in the automaton) to longer partial alignments by choosing one of several compatible extension matches (adjacent vertices to the end of a path in the automaton). To perform this search efficiently, we prune the search by computing the Chi-squared CDF and binomial CDF statistics of the partial matches and use thresholds to ensure reasonable size agreement of the matched compound fragments, and the frequency of putative missing cut sites. We conclude this section by giving an example of the backward search.

#### Size agreement

We use the Chi-squared CDF statistic to assess size agreement. This assumes the fragment size errors are independent, normally distributed events. For each pair of matched compound fragments in a partial alignment, we take the mean between the two as the assumed true length and compute the expected standard deviation using this mean. Each compound fragment deviates from the assumed true value by half the distance between them. These two values contribute two degrees of freedom to the Chi-squared calculation. Thus, each deviation is normalized by dividing by the expected standard deviation, these are squared, and summed across all compound fragments to generate the Chi-squared statistic. We use the standard Chi-squared CDF function to compute the area under the curve of the probability mass function up to this Chi-squared statistic, which gives the probability two Rmap segments from common genomic origin would have a Chi-squared statistic no more extreme than observed. This probability is compared to Kohdista’s chi-squared-cdf-thresh and if smaller, the candidate compound fragment is assumed to be a reasonable match and the search continues.

#### Cut site error frequency

We use the Binomial CDF statistic to assess the probability of the number of cut site errors in a partial alignment. This assumes missing cut site errors are independent, Bernoulli processes events. We account for all the putatively conserved cut sites on the boundaries and those delimiting compound fragments in both partially aligned Rmaps plus twice the number of missed sites as the number of Bernoulli trials. We use the standard binomial CDF function to compute the sum of the probability density function up to the number of non-conserved cut sites in a candidate match. Like the size agreement calculation above, this gives the probability two Rmaps of common genomic origin would have the number of non-conserved sites seen or fewer in the candidate partial alignment under consideration. This is compared to the binom-cdf-thresh to decide whether to consider extensions to the given candidate partial alignment. Thus, given a set of Rmaps and input parameters binom-cdf-thresh and chi-squared-cdf-thresh, we produce the set of all Rmap alignments that have a Chi-squared CDF statistic less than chi-squared-cdf-thresh and a binomial CDF statistic less than binom-cdf-thresh. Both of these are subject to the additional constraint of a maximum consecutive missed restriction site run between aligned sites of two and a minimum aligned site set cardinality of 16.

### Example traversal

A partial search for a query Rmap [3 kb, 7 kb, 6 kb] in Fig. 1a and Table (b) given an error model with a constant 1 kb sizing error would proceed with steps:

1. Start with the semi-open interval matching the empty string [0…12).
2. A wavelet tree query on ${\textsf {BWT}}$ would indicate the set of symbols {5, 6, 7} is the intersection of two sets: (a) the set of symbols that would all be valid left extensions of the (currently empty) match string and (b) the set of size appropriate symbols that match our next query symbol (i.e. 6 kb, working from the right end of our query) in light of the expected sizing error (i.e. 6kb +/− 1 kb).
3. We would then do a GCSA backward search step on the first value in the set (5) which would yield the new interval [4…7). This new interval denotes only nodes where each node’s common prefix is compatible with the spelling of our current backward traversal path through the automaton (i.e. our short path of just [5] does not contradict any path spellable from any of the three nodes denoted in the match interval).
4. A wavelet tree query on the ${\textsf {BWT}}$ for this interval for values 7 kb +/− 1 kb would return the set of symbols {7}.
5. Another backward search step would yield the new interval [8…9). At this point our traversal path would be [7, 5] (denoted as a left extension of a forward path that we are building by traversing the graph backward). The common prefix of each node (only one node here) in our match interval (i.e. [7 kb]) is compatible with the path [7, 5]. This process would continue until backward search returns no match interval or our scoring model indicates our repeatedly left extended path has grown too divergent from our query. At this point backtracking would occur to find other matches (e.g. at some point we would backward search using the value 6 kb instead of the 5 kb obtained in step 2.)

**Table 1:** Performance on simulated *E. coli* dataset

Method	Time (s)	Memory (MB)	Align-ments	Recall	Precision
Kohdista	20	19.0	907	702/4305 (16%)	702/771 (91%)
Kohdista (lax)	373	18.3	8545	3925/4305 (91%)	3925/8545 (46%)
Valouev et al.	148	4.0	742	699/4305 (16%)	699/742 (94%)
MalignerDP	47	6.0	1959	1296/4305 (30%)	1296/1959 (66%)
OMBlast	116	2078	1008	806/4305 (19%)	806/1008 (80%)
RefAligner	31	81.2	992	958/4305 (22%)	948/992 (97%)
MalignerIX	4	6.0	0	0/4305 (0%)	0/0 (N/A)
OPTIMA	455	10756.5	0	0/4305 (0%)	0/0 (N/A)

Kohdista (lax) demonstrates that our indexing and search method is capable of finding the majority of ground truth alignments when the search is pruned to the more relaxed thresholds for chi-squared-cdf-thresh and binom-cdf-thresh, i.e., chi-squared-cdf-thresh = 0.02 and binom-cdf-thresh = 0.5

### Practical considerations

In this section we describe some of the practical considerations that were made in the implementation.

#### Memoization

One side effect of summing consecutive fragments in both the search algorithm and the target data structure is that several successive search steps with agreeing fragment sizes will also have agreeing sums of those successive fragments. In this scenario, proceeding deeper in the search space will result in wasted effort. To reduce this risk, we maintain a table of scores obtained when reaching a particular lexicographic range and query cursor pair. We only proceed with the search past this point when either the point has never been reached before, or has only been reached before with inferior scores.

#### Wavelet tree threshold

The wavelet tree allows efficiently finding the set of vertex labels that are predecessors of the vertices in the current match interval intersected with the set of vertex labels that would be compatible with the next compound fragment to be matched in the query. However, when the match interval is sufficiently small ($< 750$) it is faster to scan the vertices in ${\textsf {BWT}}$ directly.

#### Quantization

The alphabet of fragment sizes can be large considering all the measured fragments from multiple copies of the genome. This can cause an extremely large branching factor for the initial symbol and first few extensions in the search. To improve the efficiency of the search, the fragment sizes are initially quantized, thus reducing the size of the effective alphabet and the number of substitution candidates under consideration at each point in the search. Quantization also increases the number of identical path segments across the indexed graph which allows a greater amount of candidate matches to be evaluated in parallel because they all fall into the same ${\textsf {BWT}}$ interval during the search. This does, however, introduce some quantization error into the fragment sizes, but the bin size is chosen to keep this small in comparison to the sizing error.

## Results

We evaluated Kohdista against the other available optical map alignment software. Our experiments measured runtime, peak memory, and alignment quality on simulated *E. coli* Rmaps and experimentally generated plum Rmaps. All experiments were performed on Intel Xeon computers with $\ge$ 16 GB RAM running 64-bit Linux.

*Parameters used* We tried OPTIMA with both “p-value” and “score” scoring and the allMaps option and report the higher sensitivity “score” setting. We followed the OPTIMA-Overlap protocol of splitting Rmaps into *k*-mers, each containing 12 fragments as suggested in [8]. For OMBlast, we adjusted parameters maxclusteritem, match, fpp, fnp, meas, minclusterscore, and minconf. For MalignerDP, we adjusted parameters max-misses, miss-penalty, sd-rate, min-sd, and max-miss-rate and additionally filtered the results by alignment score. Though unpublished, for comparison we also include the proprietary RefAligner software from BioNano. For RefAligner we adjusted parameters FP, FN, sd, sf, A, and S. For Kohdista, we adjusted parameters chi-squared-cdf-thresh and binom-cdf-thresh. For the method of Valouev et al. [12], we adjusted score_thresh and t_score_thresh variables in the source. In Table 1 we report statistical and computational performance for each method.

OMBlast was configured with parameters meas = 3000, minconf = 0.09, minmatch = 15 and the rest left at defaults. RefAligner was run with parameters FP = 0.15, sd = 0.6, sf = 0.2, sr = 0.0, se = 0.0, A = 15, S = 22 and the rest left at defaults. MalignerDP was configured with parameters ref-max-misses = 2, query-miss-penalty = 3, query-max-miss-rate = 0.5, min-sd = 1500, and the rest left at defaults.

The method of Valouev et al. [12] was run with default parameters except we reduced the maximum compound fragment length (their $\delta$ parameter) from 6 fragments to 3. We observed this method rarely included alignments containing more than two missed restriction sites in a compound fragment.

### Performance on simulated* E. coli* Rmap data

To verify the correctness of our method, we simulated a read set from a 4.6 Mbp *E. coli* reference genome as follows: we started with 1,400 copies of the genome, and then generated 40 random loci within each. These loci form the ends of molecules that would undergo digestion. Molecules smaller than 250 Kbp were discarded, leaving 272 Rmaps with a combined length equating to 35x coverage depth. The cleavage sites for the XhoI enzyme were then identified within each of these simulated molecules. We removed 20% of these at random from each simulated molecule to model partial digestion. Finally, normally distributed noise was added to each fragment with a standard deviation of .58 kb per 1 kb of the fragment. This simulation resulted in 272 Rmaps. Simulated molecule pairs having 16 common conserved digestion sites become the set of “ground truth” alignments, which our method and other methods should successfully identify. Our simulation resulted in 4,305 ground truth alignments matching this criteria. Although a molecule would align to itself, these are not included in the ground truth set. This method of simulation was based on the *E. coli* statistics given by Valouev et al. [12] and resulting in a molecule length distribution as observed in publicly available Rmap data from OpGen, Inc.

Most methods were designed for less noisy data but in theory could address all the data error types required. For methods with tunable parameters, we tried aligning the *E. coli* Rmaps with combinations of parameters for each method related to its alignment score thresholds and error model parameters. We used parameterization giving results similar to those for the default parameters of the method of Valouev et al. [12] to the extent such parameters did not significantly increase the running time of each method. These same parameterization were used in the next section on plum data.

**Fig. 2:**
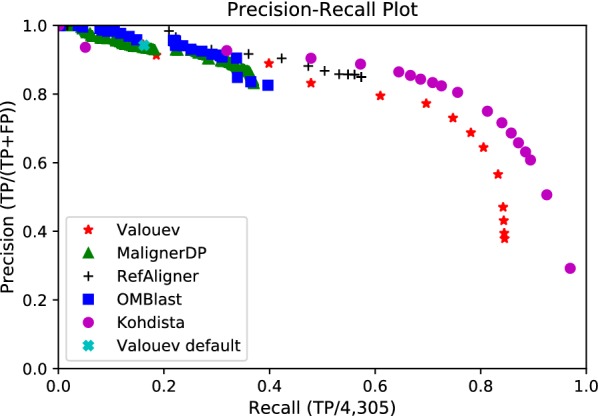
Precision-recall plot of successful methods on simulated *E. coli*

**Fig. 3:**
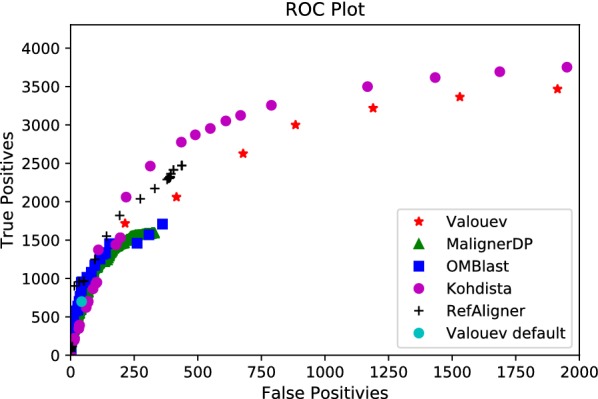
ROC plot of successful methods on simulated *E. coli*

Even with tuning, we were unable to obtain pairwise alignments on *E. coli* for two methods. We found OPTIMA only produced self alignments with its recommended overlap protocol and report its resource use in Table 1. For MalignerIX, even when we relaxed the parameters to account for the greater sizing error and mismatch cut site frequency, it was also only able to find self alignments. This is expected as by design it only allows missing sites in one sequence in order to run faster. Thus no further testing was performed with MalignerIX or OPTIMA. We did not test SOMA [13] as earlier investigation indicate it would not scale to larger genomes [14]. We omitted TWIN [14] as it needs all cut sites to match. With tuning, Kohdista, MAligner, the method of Valouev et al. [12], RefAligner and OMBlast produced reasonable results on the *E.coli* data. Results for the best combinations of parameters encountered during tuning can be seen in Figs. 2 and 3. We observed that most methods could find more ground truth alignments as their parameters were relaxed at the expense of additional false positives, as illustrated in these figures. However, only the method of Valouev et al. and Kohdista approached recall of all ground truth alignments.

Table 1 illustrates the results for Kohdista and the competing methods with parameters optimized to try to match those of Valouev et al. [12], as well as results using Kohdista with a more permissive parameter setting. All results include both indexing as well as search time. Kohdista took two seconds to build its data structures. Again, Kohdista uses Chi-squared and binomial CDF thresholds to prune the backtracking search when deciding whether to extend alignments to progressively longer alignments. More permissive match criteria, using higher thresholds, allows more Rmaps to be reached in the search and thus to be considered aligned, but it also results in less aggressive pruning in the search, thus lengthening runtime. As an example, note that when Kohdista was configured with a much relaxed Chi-squared CDF threshold of .5 and a binomial CDF threshold of .7, it found 3925 of the 4305 (91%) ground truth alignments, but slowed down considerably. This illustrates the index and algorithm’s capability in handling all error types and achieving high recall.

### Performance on plum Rmap data

The Beijing Forestry University and other institutes assembled the first plum (*Prunus mume*) genome using short reads and optical mapping data from OpGen Inc. We test the various available alignment methods on the 139,281 plum Rmaps from June 2011 available in the GigaScience repository. These Rmaps were created with the BamHI enzyme and have a coverage depth of 135x of the 280 Mbp genome. For the plum dataset, we ran all the methods which approach the statistical performance of the method of Valouev et al. [12] when measured on *E. coli*. Thus, we omitted MalignerIX and OPTIMA because they had 0% recall and precision on *E. coli*. Our results on this plum dataset are summarized in Table 2.

**Table 2:** Performance on plum

Method	Time (h)	Memory	Alignments
Kohdista	31	7.4 GB	16,109,151
Valouev et al.	678	60 MB	6387
MalignerDP	214	784 MB	1,258,328
OMBlast	151	12.3 GB	424,730
RefAligner	90	374 MB	10,039

Kohdista was the fastest and obtained more alignments than the competing methods. When configured with the Chi-squared CDF threshold of .02 and binomial CDF threshold of .5, it took 31 h of CPU time to test all Rmaps for pairwise alignments in the plum Rmap data. This represents a 21× speed-up over the 678 h taken by the dynamic programming method of Valouev et al. [12]. MalignerDP and OMBlast took 214 h and 151 h, respectively. Hence, Kohdista has a 6.9× and 4.8× speed-up over MalignerDP and OMBlast. All methods used less than 13 GB of RAM and thus, were considered practical from a memory perspective. Kohdista took 11 min to build its data structures for Plum.

**Fig. 4:**
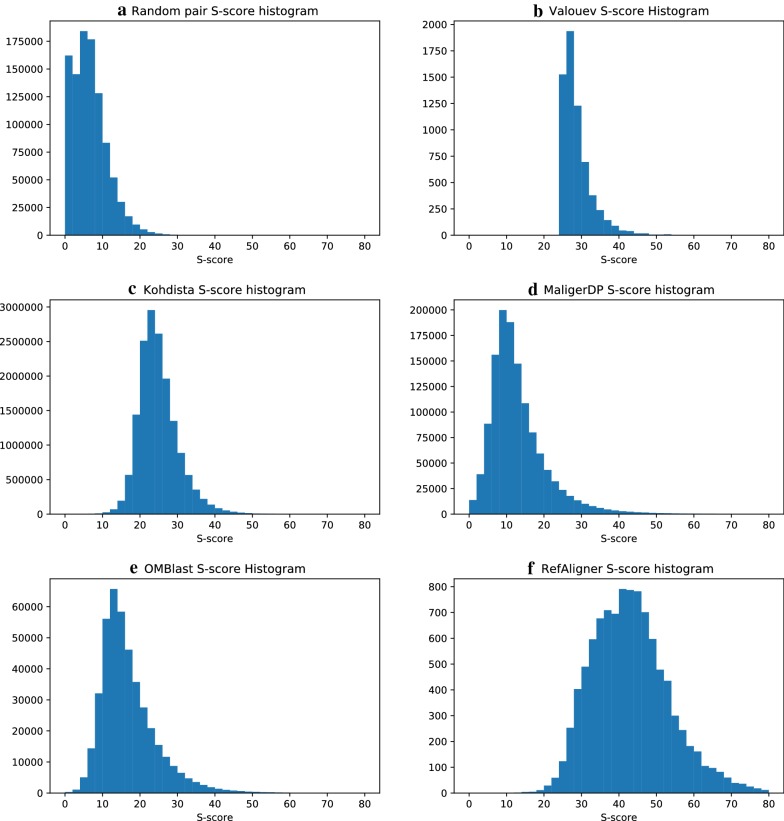
A comparison between the quality of the scores of the alignments found by the various methods on the plum data. All alignments were realigned using the dynamic programming method of Valouev et al. [12] in order to acquire a score for each alignment. Hence, the method finds the optimal alignment using a function balancing size agreement and cut site agreement known as a *S*-score. The following alignments were considered: **a** those obtained from aligning random pairs of Rmaps; **b** those obtained from the method of Valouev et al. [12]; **c** those obtained from Kohdista; **d** those obtained from MalignerDP; **e** those obtained from OMBlast; and lastly, **f** those obtained from BioNano’s commercial RefAligner

To measure the quality of the alignments, we scored each pairwise alignment using Valouev et al. [12] and presented histograms of these alignment scores in Fig. 4. For comparison, we also scored and present the histogram for random pairs of Rmaps. The method of Valouev et al. [12] produces very few but high-scoring alignments and although it could theoretically be altered to produce a larger number of alignments, the running time makes this prospect impractical (678 h). Although Kohdista and RefAligner produce high-quality alignments, RefAligner produced very few alignments (10,039) and required almost 5x more time to do so. OMBlast and Maligner required significantly more time and produced significantly lower quality alignments.

## Conclusions

In this paper, we demonstrate how finding pairwise alignments in Rmap data can be modelled as approximate-path matching in a directed acyclic graph, and combining the GCSA with the wavelet tree results in an index-based data structure for solving this problem. We implement this method and present results comparing Kohdista with competing methods. By demonstrating results on both simulated *E. coli* Rmap data and real plum Rmaps, we show that Kohdista is capable of detecting high scoring alignments in efficient time. In particular, Kohdista detected the largest number of alignments in 31 h. RefAligner, a proprietary method, produced very few high scoring alignments (10,039) and requires almost 5× more time to do so. OMBlast and Maligner required significantly more time and produced significantly lower quality alignments. The method of Valouev et al. [12] produced high scoring alignments but required more than 21× time to do.

## Availability and requirements

Project name: Kohdista.

Project home page: https://github.com/mmuggli/KOHDISTA.

Operating system(s): Developed for 32-bit and 64-bit Linux/Unix environments.

Programming language: C/C++.

Other requirements: GCC 4.2 or newer.

License: MIT license.

Any restrictions to use by non-academics: Non-needed.

## Data Availability

Kohdista is available at https://github.com/mmuggli/KOHDISTA/. No original data was acquired for this research. The simulated *E.coli* data generated and analysed during the current study are available at https://github.com/mmuggli/KOHDISTA. The plum (*Prunus mume*) dataset used in this research was acquired from the GigaScience repository http://gigadb.org/dataset/view/id/100084/File_sort/size.

## Abbreviations

DAG: directed acyclic graph (DAG)
SA: suffix array
GCSA: generalized compressed suffix array
BWT: Burrows–Wheeler Transform
